# The Mountain

**Published:** 1861-01

**Authors:** 


ART. V.—*The Mountain.* By R. M. S. JACKSON, M.D., Corresponding Member of the Academy of Natural Sciences of Philadelphia; and of the Corps of the Geological Survey of Pennsylvania, etc. etc. Philadelphia: J. B. Lippincott & Co., 1860. pp. 632. 12mo.

WHAT a combination, medley rather, have we here of science and satire, philosophy and fun, which sometimes borders on the ridiculous, and again rises into the humor of Rabelais and the quaint sentiment of Montaigne! Changing the figure, we might speak of this volume, "The Mountain," as a table, set out with dishes rich and nutrimental, and with *entremets*—side dishes—seasoned and spiced, sometimes with artistic skill, and, again, so pungent with Cayenne and mustard as to make one's mouth a little awry by lingual and palatial contortions.

Dr. Jackson's "Mountain" is made by him to represent the entire group of the Alleghany or Appalachian Mountains which extend through eleven degrees of latitude, in a direction nearly parallel to the Atlantic, and hence they are sometimes called the Atlantic range; distant from the ocean fifty to one hundred miles, and comprising a belt of from fifty to one hundred and fifty miles wide. With so vast a range of prospect and of travel, amid scenery the most diversified—mountain spires pointing to the blue empyrean; chasms which might serve as openings to the dread abyss; jutting crags and widespread ledges; endless forests, the home of animals of every size and kind, from the quick, bounding elk to the humble mole, from the eagle soaring aloft with widespread wings to the thrifty but restless wren and the ruby-throated humming-bird, eater of nectar; streams musical in their first ripples, destructive in their rapid downward descent, and carrying with them fertility when they reach the plains below, and containing in their waters a finny world,—the author, perched as he is on the summit of the Pennsylvania Alleghany Mountain, might almost imagine himself "monarch of all he surveys," and be excused if, under the exhilaration of mountain ether, he sometimes gives utterance to thoughts and feelings which to our duller perceptions in the lowlands may seem to be extravagant and in magnified proportions. He describes in a vigorous style the scenes and objects which come under his observation in the different realms of nature, and infuses into the mind of the reader a portion of his own enthusiasm while roaming through them. The very first pages, under the head of "A Word to the Subscribers of the Mountain," are so characteristic, and must so fully enlist the sympathies of the country doctor, that we cannot forbear from introducing the initial paragraph to our readers:—

"Are you a country doctor? Do you '*pull teeth?*' Are you your own cupper, bleeder, leecher? Are you your own druggist, filling out in detail your own prescriptions? Are you surgeon, midwife, general practitioner; and have you no time to get fame and money, through any of the specialties of the profession—diseases of eye, ear, lung, stomach and bowel, cerebro-spinal axis, women and children, because you are 'boy of all work,' and have to treat them all? Are you occasionally pig, cow, and horse doctor for the neighbors? Are your patients scattered over a large and diversified surface, which you must traverse on horseback, through swampy roads, wildernesses of fallen timber, traps, deadfalls of roots, and endless continuities of black forests; and do you really *go* in all sorts of weather? Are your patients so inaccessible that most of your time is consumed in merely destroying space to get to them; and have you actually *anything to do* in your profession; and when you do your work, have you five times as much trouble to get your bills as to make them at first, your patients knowing better than you do, *what you ought* to charge, and just *when they ought* to pay? Possibly, to get along, you may be trying to farm a little, endeavoring to scratch some hard patch of desolation into agricultural propriety, or bravely to make, like the hero of 'Life in the Woods,' some tough glebe 'say beans!' This is all very well, and no doubt you will have your reward in heaven. But permit me to ask you a few more questions, absurd and ridiculous as such questions may appear to common sense—and improbable as it may be to expect any answer: Are you trying to write and publish a book, in the midst of all this horror and distraction—nights of sleepless anguish, days of despair? Are you trying to repose and dream in a drum, or paint a picture seated with your easel on a log-sled hauled by a team of oxen over the rocks, roots, logs, and stumps of a spruce-pine clearing; in short, have you been trying to do in the broils of Bedlam that which, to do aright, you should have quiet, absolute repose from all care and anxiety, still nights of vision, golden mornings of ecstatic influx, and brave days of spiritual wrestling, inspired by the gentle, heroic, and loving sympathy of the living, with solemn beckonings and greetings of grace and holiness from the dead? Then, did I understand you to say that you were two hundred and fifty miles from your publishers, two hundred and fifty miles from libraries of any extent, where you could have access to books of reference and quick facilities for correcting proof, and so on? And you have really, under all these difficulties, been going through the torments of that everlasting stone-rolling of Sisyphus of 1700 ems in the page, i-dottings, t-crossings, commas stuck in by nineteen elaborate rules, colons by five, semicolons by five, periods by five, etc.?

"These interrogations you answer all in the affirmative? Well, then, on thy brow be written FOOL! for a 'thousand years in heaven cannot recompense your miserable heart' for such a blunder, or 'make you capable of one brief joy,' after such a hideous folly! 'Ah, me! miserable! which way shall I fly?'"

In another passage, in a note, the author tells us:—

"Think of a country doctor practicing medicine in two firms, making fire-brick in one, sawing lumber in another, selling drugs in another, and speculating in mountain lands and building Health Institutes on his *own hook, all at one time!*"

He adverts, in a strain half humorous, half melancholy, to the motives which induced him to adventure on the mountain; and, claiming a superiority of country over town life, he makes an ethnological remark on the two varieties of the typical Caucasian—the Celtic and the Teutonic—renews his apology, and assigns his reasons for the delay in bringing out his book.

The preface, or "Prolegomenon," is in part a narrative of the origin, and in part an explanation of the benefits of the *Sanitarium* established by Dr. Jackson at Cresson, at the very summit of the Alleghany, on the road between Philadelphia and Pittsburg, and which is now under the direction of a company chartered for the purpose. A plea for Hygeia by illustrations sentimental and physiological, set forth in a quaint fashion of language, makes up the larger part of the preface. The author indicates, at the same time, the chief heads of the subjects to be treated of in the body of the volume.

"It is obvious, then," he tells us, "that some shape of bill of fare for this soul and body was necessary to be rendered—some statement of the availableness of the mountain in all its aspects of use, health, beauty, and teaching, or worship, ecstasy, progress, and salvation." First comes the "Geology of the mountain," as "the basis or substratum of its story—the *skeleton*, the great primordial frame-work of its wondrous body. Naturally upon this would come the enveloping mineral mass, earth-cloak or integument of the same, that is, the *soil*. Then the circulating fluids would attract attention, and the story of the waters--mineral, thermal, and pure—would appear in due order of sequence. In the chain of natural causation would follow the "Flora," the plant or vegetable life clothing of the mountain, which is again dependent upon the soil, water, climate, etc., of the same—associated with all of which would arrive a chapter of its "Fauna" or animal life. A catalogue of animals, namely: mammalia, birds, reptiles, and fishes, which inhabit the mountain, or a hurried enumeration of the different orders of creation in that department would be required. Then would arrive a consideration of the ocean, of air above, upon which all created beings depend, the great medium of existence, the reservoir of vitality or life currents."

The plan thus sketched has been carried out by Dr. Jackson with a fullness of description and detail instructive in matter, and often interesting in manner. In these respects, the work will be found useful for reference by the naturalist and the physician, and it has besides large claims on the general reader. It ought to find a place in the library of every country physician, for his own delectation and the benefit of his students, who will be taught in it to commune with nature under the guidance of science. The town physician will be told in its pages of a mountain Sanitarium, and of various mineral springs to which he may send his patients, with every prospect of their regaining their lost health and strength, and learning at the same time a life-lesson that shall show the recuperative powers of pure air and pure water, and of exercise and sports which, while acting primarily on the body, exert a magical influence on the mental and spiritual faculties of our being.

These topics are enlarged on in the "Second Book," in chapters under the heads of "Æsculapius" and of "Hygeia." In the first of these, the antiquity, dignity, and functions of the true, as opposed to the spurious physician, are enforced; and unerring thrusts made at the shams and follies of the day on the pretenses to cure disease by various juggleries. We wish that room were allowed us for more extracts, to show the manner in which the author deals with some of the high-priests at their pagan altars. The following is to the point:—

"A desperate sportsman was this Hahnemann. Like the swallow-fishers on the towers of the Alhambra, flinging their flies to the wind for winged prey, he cast his lines into the vacuities of infinitude, flung out his world-wide nets of spiritual cobwebs, and, after divers hopeless rakings of the seas of space, returns with his basket crowded with shining prey: the *world's riddles* read, the sphinx's story told, mankind physically redeemed by spiritualized sugar-molecules under the divine guidance of a newly-invented force of the universe called 'similia similibus curantur.'"

"From the region of shades and mysticisms comes also this portentous humbug of the times, the cure of all diseases by water alone, this thing called Hydropathy. The modern author of this revelation is one Preissnitz, a peasant, who, being the seventh son of a seventh son, and born on Christmas night, was thus, by an eternal edict, a law of foreordination, made the recipient of that rarely condescended compliment of the supernal's creative genius."

Freshness and point abound in the short chapter on Hygeia: apt use is made of the works of Hufeland and Herbert Mayo on the occasion.

Two episodical chapters, entitled "Antæus the Giant," and "Pan, a Symbol of the Universe," terminate the work. They contain passages of no little power and eloquence on the great theme which seems to be ever present to the mind of the author, viz., man's physical regeneration and associate moral purity.

